# Editorial: The Hierarchical Organization of Supramolecular Systems: From Fundamentals to Biomedical Applications

**DOI:** 10.3389/fbioe.2021.754980

**Published:** 2021-08-30

**Authors:** Chenxuan Wang, Lei Liu, Xiaoguang Wang

**Affiliations:** ^1^ State Key Laboratory of Medical Molecular Biology, Institute of Basic Medical Sciences Chinese Academy of Medical Sciences, School of Basic Medicine Peking Union Medical College, Beijing, China; ^2^ Institute for Advanced Materials, Jiangsu University, Zhenjiang, China; ^3^ William G. Lowrie Department of Chemical and Biomolecular Engineering, The Ohio State University, Columbus, OH, United States; ^4^ Sustainability Institute, The Ohio State University, Columbus, OH, United States

**Keywords:** assembly, intermolecular interaction, biomaterials, nanotechnology, colloidal chemistry

The non-covalent interactions between small molecules, including hydrogen bonding, hydrophobic interactions, Coulombic interactions, van der Waals interactions (e.g., dipole-dipole interactions and dispersion), engender the highly specific supramolecular architectures observed among biologically relevant molecular recognition processes, such as the aggregation of lipid assemblies, the folding of DNA, RNA and protein, and the formation of membrane-less organelles. Inspired by the bottom-up design principles underlying these versatile, naturally occurred structures, a myriad of supramolecular systems were artificially constructed through a semi-empirical or *ab initio* design of the subtle, intermolecular forces among a variety of biomolecular building blocks to achieve a hierarchically assembed structure ranging from nano- to macroscale. In recent years, this research field has become a focus of a rapidly growing community integrating physics, chemistry, biology, engineering, and material science. One fascinating output emerging from this booming supramolecular chemistry and self assembly-related research is the application of supramolecular systems for engineering novel biomedical materials. The distinct biochemical and physical characteristics of supramolecular materials provide a promising opportunity for improving disease diagnosis, therapeutics, and prognosis.

This Research Topic was therefore organized to include two Original Research articles and six Review articles, which reflect recent contributions to the rational design of the hierarchical organization of supramolecular structures and new findings related to the biomedical applications of molecular assembly systems. A major initiative of this Research Topic was aimed at stimulating the broad interest of the readers of Frontiers series of journals to explore the forefront of supramolecular science and discover new potential fields of application. The contributions to this Research Topic span from the theory-based efforts that provided a profound understanding of the driving forces underlying sophisticated supramolecular architectures to the pioneering studies into the innovative biomedical application of supramolecular materials.

Diverse biomolecular architectures in nature provide abundent molecular building blocks to create hierarchical supramolecular systems, offering inspiration for engineering the next generation of biomaterials. Xuan et al. focused on hydrogen-bonded cross-β conformations which are prevalent in the supramolecular structure adopted by amyloidal proteins in nature and introduced efforts to employ β-sheet packing fibrils formed by amyloidal protein assembly as a network skeleton to frame hydrogels. The continuous stack of amyloidal protein β-sheets enhanced amyloid protein-derived hydrogels by enabling a tunable mechanical strength and an editable sticking property. These enhanced characteristics of amyloid-based hydrogels make it feasible to direct stem cell differentiation and control cell adhesion, inspiring applications in the fields of tissue repairing and cell scaffolding. Not only limited to proteins, Wang H. et al. outlined the application of natural polysaccharides-based supramolecular systems as drug delivery carriers. The ammonium or carboxylate moieties included in the saccharide units provide active binding sites that can be further crosslinked by cationic or anionic drugs, achieving the agglomeration of polysaccharides into a nanoscale carrier for drug loading.

To achieve a rational design of supramolecular architecture, substantial theoretical efforts have been contributed towards revealing the driving forces for molecular self assembly. Conventionally, supramolecular assembly structures are conceptually thought to be governed by an energetic summarization of the intermolecular interactions among biomolecular building blocks. In contrast to the conventional opinion, Yu et al. point out the necessity to consider the interplay among different types of non-covalent interactions, and investigated the regulatory effects of immobilized cations on the folding and assembly structure of melittin, a globally amphiphilic cell-penetrating peptide composed of cationic residues, lysine (Lys) and arginine (Arg). By comparing the homozygous mutants of melittin that exclusively contain either immobilized ammonium (melittin-Lys) or immobilized guanidinium (melittin-Arg), they demonstrate that the chemical identity of cationic residues modulates the hydrophobically driven melittin self association and biological activity.

The chirality of amino acids has been recognized to be essential for peptide assembly structures. Zheng et al. presented advances in the understanding of the regulatory effects of chirality substitution on the structure of peptide assemblies. The outcomes caused by the changing of the chirality of amino acids in self assembled peptide sequences from L to D-configuration are not only limited to distorting the peptide backbone and disrupting the ordering of peptide packing but also provide an unexpected opportunity where heterochiral peptides self-assemble or co-organize themselves in a divergent supramolecular pattern that is distinct from homochiral peptide assemblies. Beyond the chiral self-sorting effect, the mechanisms underlying the intermolecular recognition among the heterochiral peptides or racemic peptide enantiomers remain unknown and require further explorations of the effects of chirality alteration on their assembly propensity.

The interface formed between biomolecules and inorganic materials is another critical factor that influences the application of assemblies. Liu et al. reviewed the approaches used to engineer the interface between nanomaterials and conjugated peptide ligands. Chemical modification of nanosized drug carriers with receptor-targeting drug ligands has emerged as an important strategy to pursue improved drug target specificity and effective accumulation. The biological activity of a drug-modified nanomaterial co-assembly displays non-additive characteristics, in which the interface formed between the drug ligands and nanomaterials enhances the co-assembled system with distinct properties for drug delivery. The authors illustrated a synergistic effect between the nanosized structures and receptor-targeting peptides on receptor-peptide ligand binding specificity, strengthening active targeting tumor therapy.

The versatile structures and properties of supramolecular systems motivate the emergency of supramolecular materials in clinical applications, improving disease diagnosis and treatment. Cheng and Li synthesized an amphiphilic building block (N, N′-diaspartate-3, 4, 9, 10-perylene tetracarboxylic acid imide, NAAPD) by conjugating an aromatic perylene diimide with two anionic aspartic acids. Physical stimuli, such as ultrasound, can be utilized as a trigger to facilitate the formation of intermolecular π-π packing interactions between the aromatic moiety of perylene diimide, leading to a stimuli-responsive NAAPD hydrogenation. The NAAPD-derived hydrogel displays a promising potential as a drug delivery platform that can achieve a controlled release of an anti-cercariae drug. In addition, Wang Z. et al. summarized current strategies for microbubble-based thrombus targeting. Microbubbles with an enhanced ultrasound responsiveness have attracted growing attention due to their potential in the treatment of thrombotic diseases. Coating microbubbles with high affinity, small molecular ligands which can specifically recognize the epitopes located at activated platelets and fibrin enables microbubbles with the capability of active targeting and increased accumulation of drugs at the site of thrombus. Hao et al. summarized recent advances in the design and manufacturing of supramolecular systems for carrying glucocorticoid drugs. Glucocorticoids are anti-inflammatory reagents possessing potential as a treatment against rheumatoid arthritis, asthma, atopic dermatitis, allergic rhinitis, and chronic rhinosinusitis. The side effects of glucocorticoids causing hypothalamic–pituitary–adrenal suppression-related complications, however, prevent the clinical practice of glucocorticoids. It is believed that supramolecular drug delivery platforms may provide a satisfactory solution to overcome the challenge of glucocorticoids.

In conclusion, this specific Research Topic provides an overview of some of the most fascinating advances in the current research of the biomedical applications of supramolecular materials. We hope this Research Topic can inspire the interdisciplinary integration of the pioneering research between researchers in the field of supramolecular science and self assembly, further promoting the growth of this rapidly booming discipline. It should be noted that studies in this field are still in a preliminary stage. The level of structural complexity of self assembly of synthetic low molecular weight molecules achieved in the lab is still impressively simple compared with the naturally occurred assembled systems. More efforts are needed for the future study to reveal the mystery left in the natural biomolecular structures and provide informative instructions for creating hierarchical, multifunctional structures and guiding their behaviors more like nature.

